# Development and Preliminary Testing of App-Based Culturally-Adapted Psychoeducation for Bipolar Disorder in Pakistan

**DOI:** 10.1192/bjo.2024.110

**Published:** 2024-08-01

**Authors:** Muqaddas Asif, Ameer B. Khoso, Nasim Chaudhry, Imran B. Chaudhry, Muhammad Ishrat Husain

**Affiliations:** 1 Pakistan Institute of Living and Learning, Karachi, Pakistan; 2 University of Manchester, Manchester, United Kingdom; 3 Ziauddin University, Karachi, Pakistan; 4 Centre for Addiction and Mental Health, Toronto, Canada; 5 University of Toronto, Toronto, Canada

## Abstract

**Aims:**

Bipolar disorder (BD) leads to marked disability, morbidity, and premature death. Although pharmacological agents are an essential part of BD treatment, psychosocial interventions have played an important role in enhancing treatment adherence, functioning and quality of life in patients with BD. Building on a successful pilot randomised controlled trial (RCT) of a Culturally adapted PsychoEducation (CaPE) intervention for BD, CaPE is currently being evaluated in a large multicenter RCT for its clinical and cost-effectiveness across Pakistan. However, innovations are urgently needed due to limited human resources and disproportionately high clinical needs to bring effective interventions to scale. This study aims to develop and test a mHealth iteration of CaPE, digital CaPE (dCaPE), to be delivered via a mobile app.

**Methods:**

The study will utilise a two-phased approach to i) develop a user-centred dCaPE mobile application and ii) assess the feasibility and preliminary efficacy of dCaPE for people with BD in a randomised controlled trial in Pakistan. For application development, we have conducted discussion groups with stakeholders i.e., mental health professionals (psychiatrists, psychologists, nurses) (n = 8) and patients and carers (n = 10) to gauge their valuable insights for app design, visual elements, cultural sensitivity, motivational and mood-monitoring features, and app functionality to improve user experience.

**Results:**

The findings from discussion groups informed the importance of visual elements, specifically font size and style. Participants recommended the use of soft and soothing colours like white, grey, and soft shades of pink to prevent overstimulation. Additionally, participants highlighted the need for culturally and linguistically inclusive features, including emojis and audio messages for effective engagement and to address the challenge of low literacy. The mHealth approach was deemed highly valuable, especially given the prevalence of mental health challenges and associated stigma. Endorsed by participants, the dCaPE application will offer customized psychoeducation messages along with daily 5-item (mood, energy, sleep, medication, and irritability) screening, a weekly comprehensive test for manic and depressive episodes based on DSM–5 criteria; weekly reminders to regulate sleep and eating habits, and visual representations of weekly mood monitoring reports with the incentive of badges or rewards for goal achievers.

**Conclusion:**

This research has the potential to enhance clinical outcomes, social and occupational functioning, and the overall quality of life for BD patients while addressing substantial mental health treatment gaps with impact and implications extending to various low-resource settings.

